# Stopping, opening, and redirecting fluid flow in paper-based microfluidic devices using thermo-responsive gelatin valves

**DOI:** 10.1039/d6ra07964d

**Published:** 2026-10-05

**Authors:** Abdolali Mehrjou, Seyed Hamid Safiabadi Tali, Mohsen Ghiasi Tarzi, Zubi Sadiq, Sana Jahanshahi-Anbuhi

**Affiliations:** a Department of Chemical and Materials Engineering, Gina Cody School of Engineering, Concordia University Montréal Québec H4B 1R6 Canada sana.anbuhi@concordia.ca

## Abstract

The advancement of microfluidic paper-based analytical devices (µPADs) toward complex biochemical assays is hindered by the lack of reliable, low-cost fluid-control strategies capable of providing defined sealing and actuation times. The environmental sensitivity of passive barriers depends on their material composition and formulation, whereas many active valves require comparatively complex fabrication. This work introduces a thermo-responsive gelatin valve as a simple, inexpensive, and biocompatible approach to fluid control. Valve performance was optimized by varying gelatin concentration, cooling conditions, storage duration, and actuation temperature. A concentration of 30 mg mL^−1^ provided the most suitable balance between closed-state retention and thermal responsiveness. Porometry and contact-angle measurements indicated that the gelatin-treated region reduced accessible capillary pathways and exhibited a water contact angle of approximately 99°, thereby preventing flow at room temperature. Under controlled heating at 40 °C, the optimized valve opened in approximately 20 s. The valve was integrated into a paper-based acetylcholinesterase inhibition assay for malathion. A single-shot design reduced the number of liquid-addition steps by combining sample delivery and incubation in one operation. The calculated limit of detection (LOD) was 0.24 µM, below the drinking-water guideline value of approximately 0.88 µM. A 0.5 µM malathion spike in St. Lawrence River water produced a recovery of 86.1 ± 3.2%. The device is presented as a proof-of-concept screening platform, with further matrix validation required before regulatory drinking-water analysis. These findings establish gelatin as a tunable material for timed fluid control in paper microfluidic devices.

## Abstract 2

Directly deposited gelatin valves enable on-demand fluid control in paper microfluidic devices. Controlled heating at 40 °C opens the valve, allowing timed reagent delivery for multistep assays.

## Graphical abstract

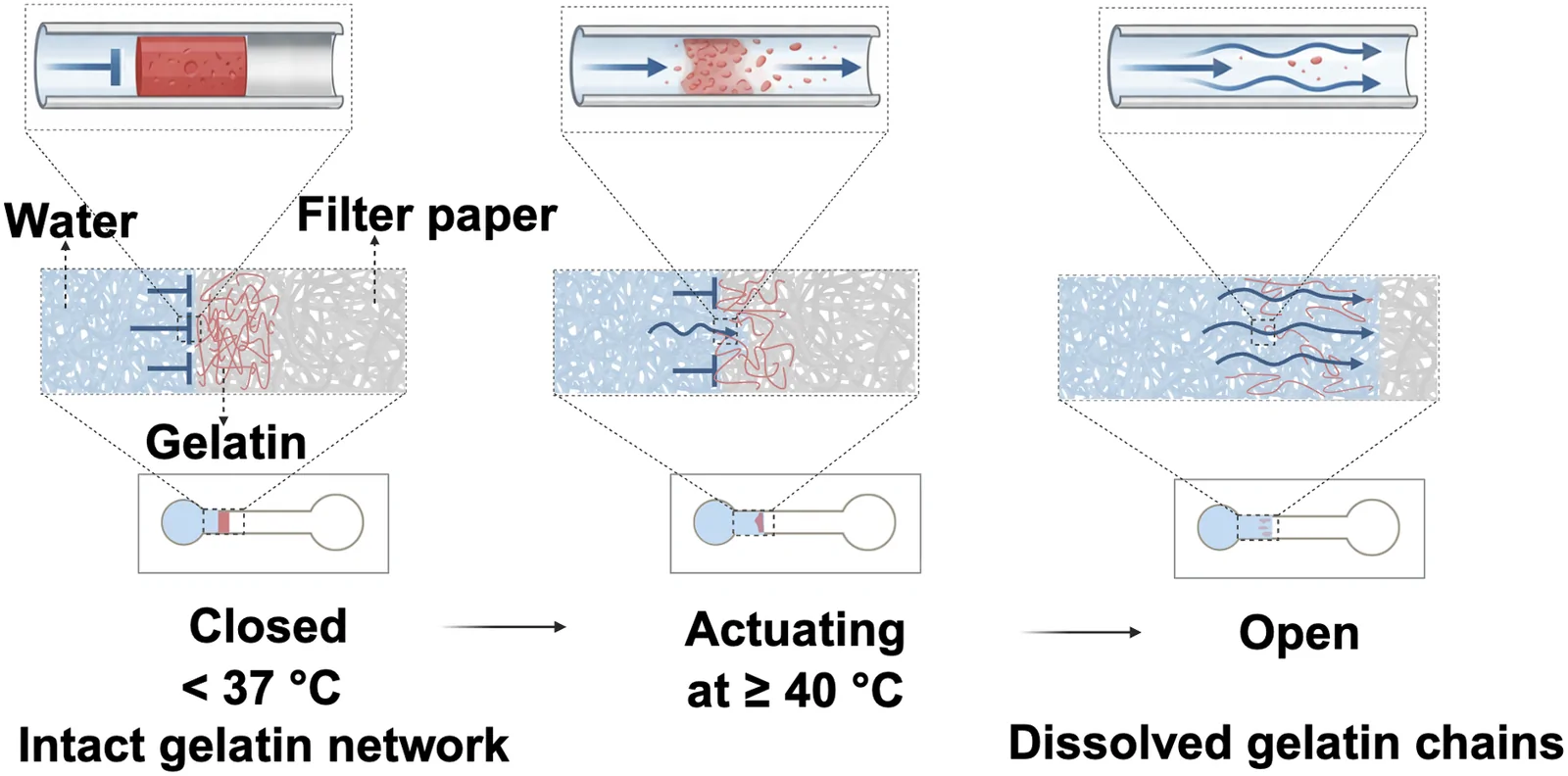

## 1 Introduction

Microfluidic paper-based analytical devices (µPADs) have become an important class of point-of-care (POC) assays since they combine low cost, simple operation, and passive capillarity flow with the potential for mass production.^1^ Since the original demonstrations of paper assays, the µPAD field has advanced into a comprehensive set of fabrication methodologies and device architectures for medical diagnostics, environmental monitoring, and food-safety testing.^2^ The passive, pump-free nature of paper, where liquids move by capillary forces through hydrophilic channels, gives µPADs a unique advantage for resource-limited settings. However, there are challenges for multi-step biochemical reactions that require precise incubation time and ordered sample delivery.^3^ Reliable timing and sequential sample delivery are essential for many enzyme-based and multi-reagent assays (*e.g.*, inhibition assays, multistep colorimetric methods, enzyme-linked reactions) because reactions order and incubation times directly affect sensitivity, specificity, and reproducibility.^4^ Researchers have developed a broad toolbox of passive and active approaches for flow control in µPADs to address this issue. Passive approaches include channel geometry design (*e.g.*, gravity-driven,^5^ fast/slow channels,^6^ capillary delay elements^7^), dissolvable or erodible barriers,^8,9^ and capillary valving strategies.^10^ Thermally activated wax valves,^9,11^ shape-memory polymers,^12,13^ pneumatic and centrifugal actuation,^14^ and integrated microheaters or electronics^15^ are among active methods.

Each flow-control strategy offers trade-offs between fabrication and integration complexity, operational simplicity, tunability, and susceptibility to environmental conditions.^16^ Among passive valves, dissolvable or erodible polymer films and sugar-based barriers have been attractive in µPADs because they enable time delays without external hardware: a bridge of dissolvable material blocks flow until it erodes away, after which the downstream channel floods.^17^ These methods are simple and inexpensive, and their timing can be tuned by barrier composition and thickness. Although dissolvable or erodible barriers provide passive fluid control without requiring external actuation, their sensitivity to environmental humidity depends on the chemical composition, geometry, thickness, and packaging conditions. Hygroscopic formulations may exhibit humidity-dependent dissolution or delay times during storage, whereas less hygroscopic materials can provide improved environmental stability.^18,19^ Similarly, geometric approaches (fast/slow channels) avoid additional materials but generally provide only short delays and are strongly affected by paper lot-to-lot variability and environmental conditions.^20^ These limitations motivate the exploration of alternative active-valve materials and mechanisms that provide user-defined temporal control while maintaining simple fabrication.

Hydrogels and polymeric gels have increasingly been integrated into microfluidic systems as functional materials for sensing, controlled release, and actuation because they can be engineered to respond to stimuli (pH, temperature, ionic strength) while remaining biocompatible.^22,23^ Strategies for integrating functional materials and surface-modification techniques into polymeric and paper microfluidic systems emphasize two benefits relevant to valve design: (i) material-level tuning of response characteristics^24,25^ (*i.e.*, the gelation temperature, crosslink density, and swelling behavior) and (ii) relatively low-barrier fabrication methods. Embedding responsive gels directly into channels enables stimulus-responsive gating and timed fluid release,^26^ but the literature also points out practical concerns such as material-substrate adhesion, mechanical robustness on porous substrates, and changes in microstructure upon drying and storage, which must be characterized experimentally for each gel–substrate combination.^27^

Early research largely focused on synthetic polymers such as poly(*N*-isopropylacrylamide) (PNIPAM), which exhibits a sharp volume-phase transition near physiological temperatures.^28,29^ PNIPAM-based soft valves have been engineered for rapid and reversible fluidic switching.^30^ Other approaches have used thermally responsive composites, expandable microspheres in PDMS, dual-responsive hydrogel films, and integrated thermal relay valves.^31–33^ Despite these advances, response time, robustness, fabrication complexity, and compatibility with biochemical reagents remain important design considerations.^22,34^ Active valves that respond at mild temperatures may reduce the thermal load applied to sensitive assay reagents and facilitate operation using compact external heaters.

In this work, for the first time, we investigate gelatin as a thermo-responsive valve material for µPADs. Gelatin is a denatured collagen protein that forms physical gels during cooling through partial helix reformation and returns to a liquid-like sol state upon moderate heating.^35^ Unlike valve materials that require polymer synthesis, grafting, or mechanically integrated components, gelatin is commercially available and can be deposited directly into paper channels without any modification.^22^ Its response can be tuned through concentration and cooling conditions.^36^ We characterize valve retention, actuation, storage behavior, wettability, accessible pore pathways, and morphology. As a proof-of-concept, the valves were integrated into a two-step AChE inhibition assay for malathion. The single-shot design reduced manual liquid additions by combining sample delivery and incubation, while controlled heating released the substrate after the defined incubation period. These results position gelatin as a tunable timing element for multi-step paper assays, while the limitations associated with environmental temperature, humidity, storage, and single-use operation are explicitly acknowledged.

## 2 Experimental section

### 2.1. Chemicals and materials

Gelatin (type A, from porcine skin, Cat. no. G1890), malathion (Cat. no. 36143), indoxyl acetate (Cat. no. I3500), and acetylcholinesterase (Cat. no. C3389) were purchased from Sigma-Aldrich. Tris(hydroxymethyl) aminomethane (Cat. no. TB0194) was purchased from Bio Basic. Whatman grade 1 filter paper (Cat. no. WHA1001917) was purchased from Fisher Scientific, Canada. Parafilm^®^ M (Amcor PLC, Cat. no. PM-996) was purchased from central chemical store at Concordia University, Canada. Neon food colors (Clubhouse) were purchased from a local Walmart store, Canada. Deionized water was used throughout.

### 2.2. Fabrication of microfluidic paper-based analytical devices (µPADs)

The fabrication of µPADs was done based on Parafilm^®^-heating-laser-cutting (PHLC) described in our previous work.^37^ Whatman grade 1 filter paper was used for all µPADs. Briefly, a sheet of Parafilm^®^ M is placed underneath the filter paper (acting as a hydrophobic backing). The stack is heated (*e.g.*, in an oven or hot plate) to thermally bond the Parafilm^®^ to the paper without fully saturating the fiber network, preserving the paper's hydrophilicity. Then, a CO_2_ laser cutter is used to selectively ablate through the paper layer. The laser cutter defines the boundaries of the microfluidic channels. These hollow microstructures act as impenetrable hydrophobic barriers, guiding the fluid flow within the un-cut hydrophilic paper zones. The patterns on µPADs were designed using Inkscape software version 1.1.1. Details of the laser settings are provided in Table S1.

### 2.3. Fabrication of gelatin valves

Fig. 1 illustrates fabrication of the gelatin valve on the µPAD. Gelatin solutions at 10, 20, 30, 35, 40, and 45 mg mL^−1^ were prepared in deionized water. Each solution was mixed and heated at 65 °C for 10 min, then mixed again immediately before deposition. For visualization experiments, one drop of red food dye was added to the gelatin solution and blue-dyed water was used as the test fluid. A 0.5 µL aliquot of gelatin solution was deposited onto the designated valve region. The devices were then cooled either at ambient temperature or under refrigerated conditions, as described below.

**Fig. 1:**
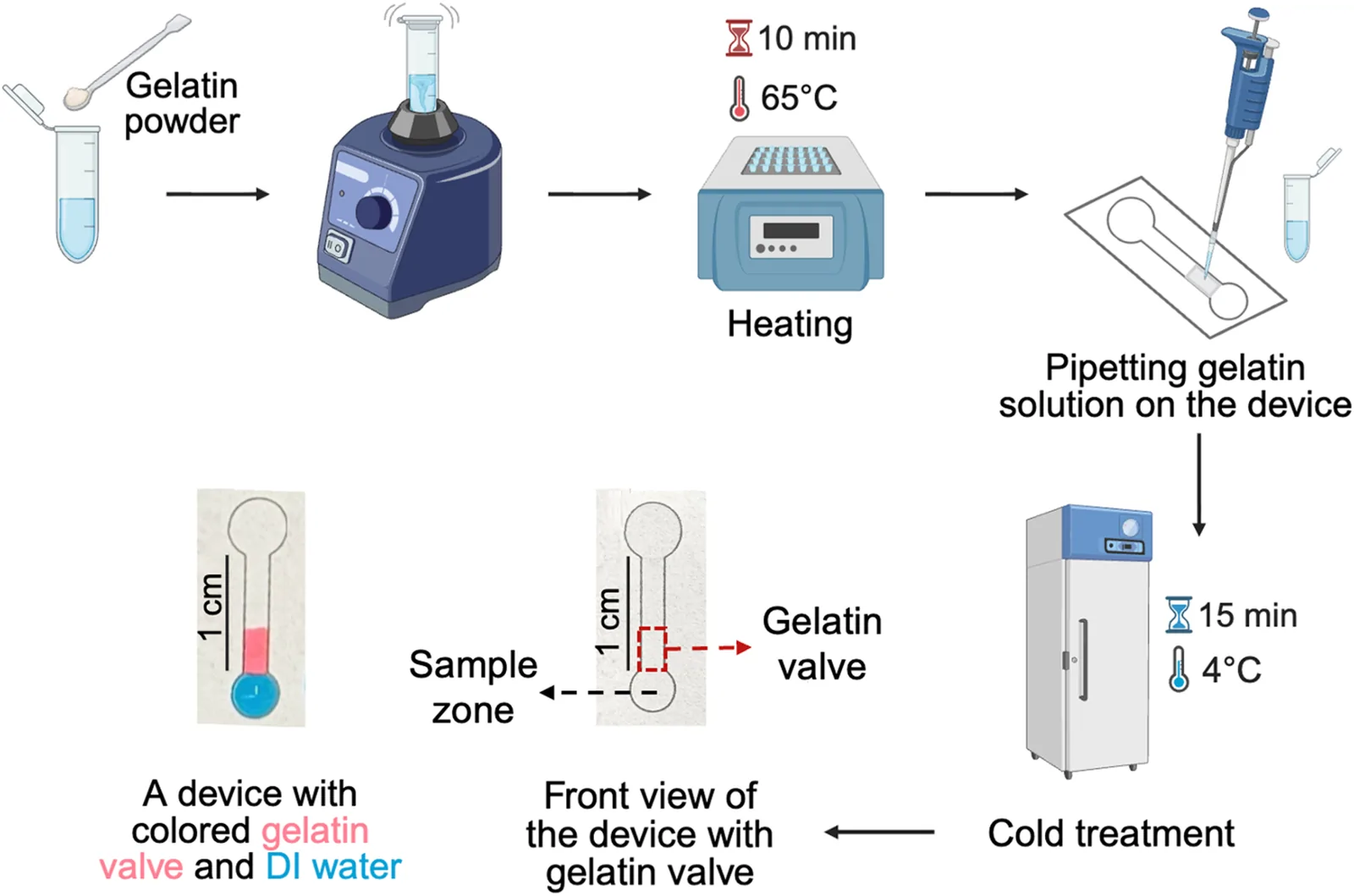
Schematic of preparing a device with gelatin valve for characterization tests.

### 2.4. Gelatin valve optimization

The valves were gelled at either ambient temperature (20 ± 2 °C) or 4 °C. To assess cooling time and temperature, devices containing 30 mg mL^−1^ gelatin valves were cooled for 5, 10, or 15 min. After cooling, 7 µL of blue-dyed water was added to the inlet and fluid progression was monitored for up to 35 min. A condition was considered to provide reliable closed-state retention only when all four independently prepared valves remained closed throughout the observation period. Each condition was tested in quadruplicate.

Gelatin concentrations from 10 to 45 mg mL^−1^ were evaluated by adding 7 µL of dyed water at 20 ± 2 °C and monitoring fluid penetration for up to 35 min. Thermal actuation was evaluated for valves prepared with 30, 35, 40, and 45 mg mL^−1^ gelatin. Devices containing 7 µL of dyed water were placed in direct contact with a hot plate set between 37 and 60 °C. Actuation time was defined as the interval from application of the thermal stimulus until the fluid front completely traversed the approximately 5 mm-long gelatin-treated region (*n* = 4). For storage testing, freshly fabricated 30 mg mL^−1^ valves were sealed in zip bags and stored either at ambient conditions (20 ± 2 °C, approximately 50–60% RH) or at 4 °C. All devices used for the storage study were fabricated from the same batch and subsequently divided into independent subsets for each storage condition and evaluation time point. Closed-state retention at 20 °C and thermal actuation at 40 °C were evaluated after one week, one month, and two months and compared with freshly prepared controls.

### 2.5. Surface and pore-structure characterization

#### 2.5.1. Contact angle measurements

Water contact angles were measured using a Dropometer instrument (Droplet Lab Inc., Canada). A 3 µL droplet of deionized water was deposited on untreated paper, the 30 mg mL^−1^ gelatin-valve region before actuation, and the valve region after thermal actuation and fluid passage. Images were analyzed in ImageJ using the Drop Analysis plugin.

#### 2.5.2. Capillary-flow porometry

The pore-size distribution was measured using a capillary-flow porometer (Anton Paar Quantachrome 3Gzh). Circular Whatman grade 1 paper specimens, 25 mm in diameter, were prepared. A 50 µL aliquot of gelatin solution at 10, 100, or 1000 µg mL^−1^ was deposited at the center of each specimen to cover the test area, and the samples were cooled at 4 °C for 15 min. Untreated paper served as the control. Samples were saturated with Porofil^®^, and air pressure was gradually increased to displace the wetting liquid from connected pores. Pore diameter was calculated using the Young–Laplace relationship 
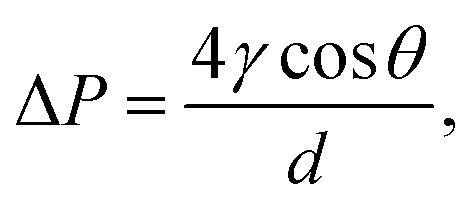
 where Δ*P* is the applied pressure difference, *γ* is the surface tension of the wetting liquid, and *θ* is the contact angle (assumed to be 0° for the wetting fluid). The measurements therefore represent accessible, continuous through-pore pathways rather than every physical void within the paper matrix.

#### 2.5.3. Scanning electron microscopy

Untreated paper, the gelatin-treated valve region before actuation, the valve region after thermal actuation and fluid passage, and the downstream detection zone were examined by scanning electron microscopy. Representative images were interpreted qualitatively because the observed morphological differences were heterogeneous. Additional SEM imaging was performed on paper treated with 45 mg mL^−1^ gelatin before and after thermal actuation to examine morphology at a higher gelatin loading.

### 2.6. Malathion detection

Malathion detection was based on inhibition of acetylcholinesterase (AChE). Active AChE hydrolyzes indoxyl acetate (IDA), leading to formation of a blue indigo product. Increasing malathion concentration suppresses AChE activity and therefore decreases the measured color intensity.^38^

AChE was prepared at 30 U mL^−1^ in 100 mM Tris buffer at pH 8. A 100 mM IDA stock solution was prepared in methanol and diluted immediately before use with 100 mM Tris buffer at pH 8 to obtain a 20 mM working solution. Malathion standards from 10 nM to 10 mM were prepared in Tris buffer containing 5% methanol and were used after preparation. Malathion standards were handled in a fume hood using appropriate personal protective equipment (PPE).

For each device configuration, 1 µL of AChE solution at 30 U mL^−1^ was deposited onto the 3 mm-diameter detection zone, and 1 µL of the 20 mM IDA working solution was deposited onto the substrate zone. The deposited reagents were air-dried at 20 °C for 10 min before use. Each device contained a gelatin valve prepared using 0.5 µL of 30 mg mL^−1^ gelatin and gelled at 4 °C for 15 min.

The assay principle, device configurations, and reagent-deposition scheme are illustrated in Fig. 2. In both configurations, the gelatin valve was used to temporally separate the AChE-inhibition step from subsequent delivery of IDA.

**Fig. 2:**
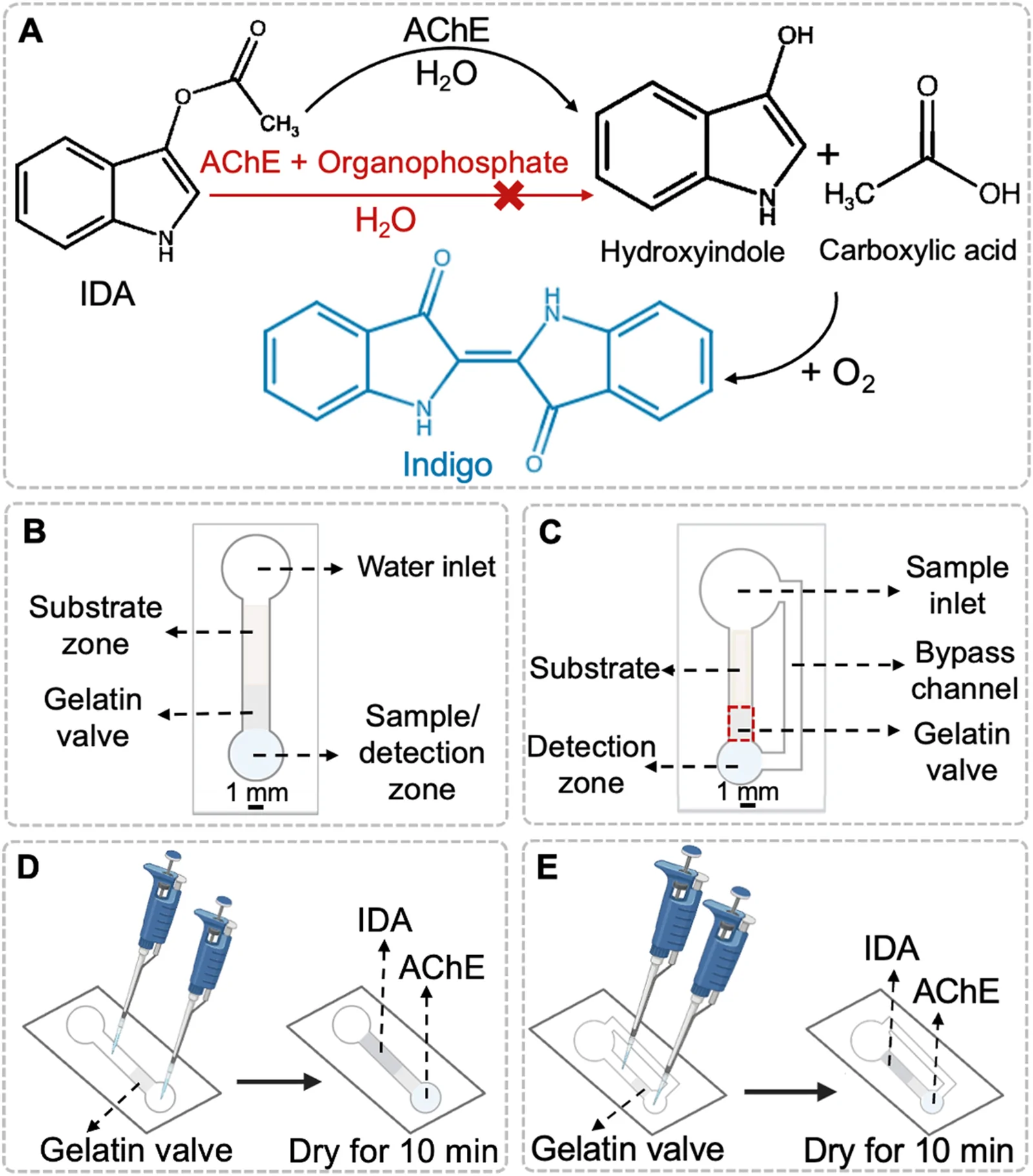
Design and working principle of the malathion-detection device. (A) AChE catalyzes IDA hydrolysis and formation of a blue indigo product; malathion inhibits this response. (B) Double-shot layout containing a water inlet, substrate zone, gelatin valve, and detection zone. (C) Single-shot layout containing a sample inlet, bypass channel for passive sample delivery, substrate zone, gelatin valve, and detection zone. (D and E) Deposition and drying of AChE and IDA in the double-shot and single-shot devices, respectively.

#### 2.6.1. Double-shot assay procedure

For the double-shot configuration, 2 µL of malathion sample was applied directly to the AChE-containing detection zone and incubated for 15 min at 20 ± 2 °C. After incubation, 7 µL of deionized water was added to the upstream inlet. The device was placed on a hot plate set to 40 °C until the fluid front completely traversed the gelatin-treated region and transported rehydrated IDA to the detection zone.

#### 2.6.2. Single-shot assay procedure

For the single-shot configuration, 15 µL of malathion sample was added to the sample inlet. The sample travelled through the bypass channel to the AChE-containing detection zone while the gelatin valve remained closed. After a 15 min inhibition period at 20 ± 2 °C, the device was placed on a hot plate set to 40 °C until the fluid front completely traversed the gelatin-treated region and delivered IDA to the detection zone.

#### 2.6.3. Image acquisition and analytical evaluation

Devices were scanned using a Canon Pixma TS5020 desktop scanner at 600 dpi in RGB mode. All devices were scanned using identical settings. Images were analyzed using ImageJ version 1.54 p. A fixed circular region of interest corresponding to the 3 mm-diameter detection zone was used, and the mean grayscale intensity was recorded. Six independently prepared devices were analyzed at each malathion concentration, and results are reported as mean ± standard deviation.

Dose–response data were fitted using the four-parameter logistic model *y* = *d* + (*a* − *d*)/[1 + (*x*/*c*)^*b*^], where *y* is grayscale intensity, *x* is malathion concentration in mol L^−1^, *a* and *d* are the upper and lower asymptotes, *b* is the slope parameter, and *c* is the half-maximal inhibitory concentration (IC_50_). Because the signal decreased as malathion concentration increased, the LOD response threshold was calculated as *y*_LOD_ = *y*_blank_ − 3(SD_blank_). The corresponding malathion concentration was obtained by inverse prediction from the fitted four-parameter logistic calibration curve and was reported as the LOD.

#### 2.6.4. Real-sample analysis

Water was collected from the St. Lawrence River in Montréal, Québec, Canada. Aliquots were spiked to final malathion concentrations of 0.0005, 0.05, and 5 mM and analyzed using the single-shot procedure. An unspiked river-water sample was used as the matrix blank. Percent recovery was calculated. These measurements were treated as exploratory matrix-response checks rather than full quantitative validation.

## 3 Results and discussion

### 3.1. Gelatin valve fabrication and optimization

Gelatin valves were fabricated by exploiting the reversible temperature-dependent transition between sol and gel states. As illustrated in Fig. 1, heated gelatin solution was deposited onto the paper channel and allowed to penetrate the cellulose network. Cooling promoted formation of a physical gelatin network that prevented fluid passage through the treated region. The fabrication parameters were optimized to provide at least 15 min of closed-state retention for the malathion assay while allowing rapid opening under controlled heating.

#### 3.1.1. Effects of cooling conditions

Cooling conditions influenced valve performance (Fig. 3A). Valves prepared at ambient temperature leaked within approximately 4 min. Refrigerated cooling reduced fluid penetration, but the 5 and 10 min conditions did not meet the predefined all-replicate retention criterion. Valves cooled at 4 °C for 15 min remained closed throughout the 35 min observation period in all tested devices, exceeding the 15 min incubation requirement of the malathion assay. Accordingly, cooling at 4 °C for 15 min was used for all subsequent experiments. The photographic series and replicate-level interpretation are provided in Fig. S1. The enhanced robustness in stopping the liquid flow is attributed to the kinetics of triple-helix formation; rapid cooling at 4 °C promotes faster helix reformation and a higher density of junction zones, resulting in a mechanically denser network compared to the loosely packed structures formed at room temperature.^39,40^

**Fig. 3:**
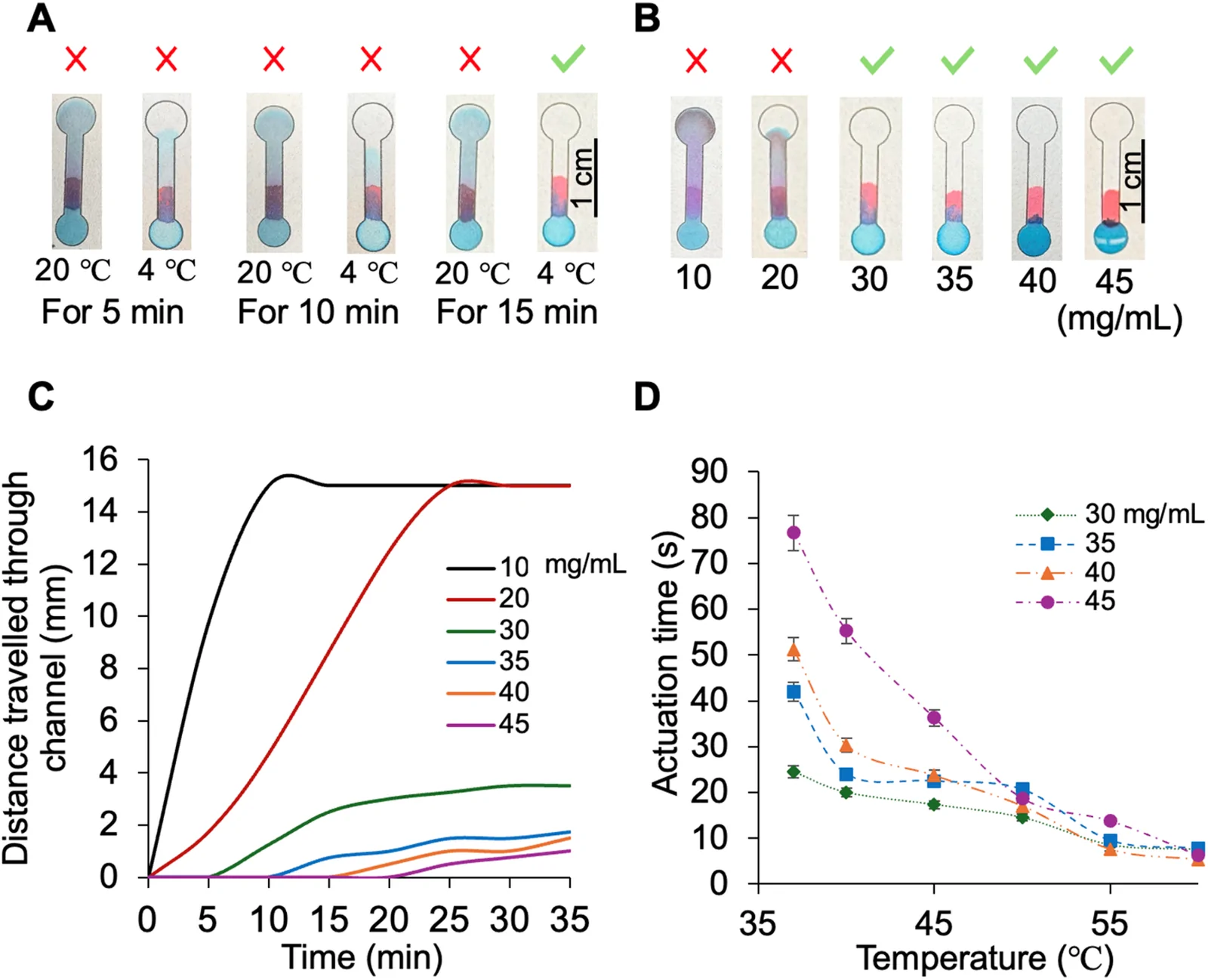
Optimization of gelatin-valve fabrication parameters. (A) Effect of cooling conditions. Only valves cooled at 4 °C for 15 min met the predefined criterion of remaining closed for the full 35 min observation period in all replicates. (B) Effect of gelatin concentration on closed-state retention. Concentrations of 30 mg mL^−1^ or greater prevented visible fluid passage throughout the observation period. (C) Distance travelled by the fluid front as a function of time. (D) Actuation time as a function of heating temperature for valves prepared with 30–45 mg mL^−1^ gelatin (mean ± SD; *n* = 4). Actuation time was measured from application of heat until the fluid front completely traversed the approximately 5 mm-long gelatin-treated region.

#### 3.1.2. Effect of gelatin concentration on valve performance

The sealing efficiency and thermal responsiveness of the valves were strongly dependent on gelatin concentration. We evaluated valves fabricated with concentrations ranging from 10 to 45 mg mL^−1^ to identify the optimal concentration capable of withstanding the assay incubation period (Fig. S2). As shown in Fig. 3B, the 10 mg mL^−1^ valves failed within approximately 5–8 min, whereas the 20 mg mL^−1^ valves delayed flow but did not maintain a reliable seal throughout the full assay period. This failure is quantitatively analyzed in Fig. 3C, which plots the distance of fluid travel inside the channel over time for the valves with different gelatin concentrations. The steep slopes observed for 10 and 20 mg mL^−1^ indicate a high flow rate, reflecting the rapid breakdown of the sparse polymer network due to insufficient crosslinking density.

In contrast, concentrations of at least 30 mg mL^−1^ were sufficient to halt fluid flow. As evidenced by the plateau in Fig. 3C, the flow rate dropped significantly for all concentrations greater than 30 mg mL^−1^, with fluid penetration restricted to negligible levels over the 35 minute observation window. While higher concentrations also provided effective sealing, 30 mg mL^−1^ was selected as the optimal concentration for our application. It offered the necessary sealing to reliably withstand the 15 minute incubation step without the excessive polymer density of higher concentrations, striking a balance between robust sealing and efficient thermal actuation.

#### 3.1.3. Thermal actuation

Thermal actuation occurred through a gel-to-sol transition. At room temperature, the valve region contained a physical gelatin network stabilized by non-covalent interactions. Heating disrupted this network, increased gelatin mobility, and permitted the fluid front to traverse the treated paper region.^41^

Fig. 3D shows the actuation kinetics as a function of applied temperature and gelatin concentration. Actuation time was defined as the interval from application of heat until the fluid front completely traversed the approximately 5 mm-long gelatin-treated region. Increasing temperature shortened actuation time at all tested concentrations. Although actuation was observed at 37 °C, the response was slower and a reliable actuation by natural body heat was not evaluated. The mild operating range is therefore considered advantageous primarily for use with compact, controlled external heaters. Ambient temperatures approaching the actuation range may reduce closed-state retention or cause premature opening; the maximum environmental operating temperature was not systematically established in this study. Gelatin undergoes a reversible sol–gel transition at the material level, but functional re-closing after fluid passage was not evaluated, and the integrated paper valve is therefore considered single-use.

Furthermore, the data highlights a controllable trade-off between valve stability and responsiveness. At lower temperatures (37–45 °C), increasing the gelatin concentration progressively prolonged actuation times due to the higher density of inter-chain junction zones requiring dissociation. Conversely, at elevated temperatures (>55 °C), thermal energy was sufficient to rapidly overcome network forces regardless of concentration, resulting in uniformly fast actuation (<20 s). This tunable behavior allows valve performance to be tailored to specific assay requirements, balancing the need for robust sealing during incubation with the desire for rapid actuation during detection steps.

The optimized condition was 30 mg mL^−1^ gelatin cooled at 4 °C for 15 min. Heating at 40 °C reduced the average actuation time to approximately 20 s (Fig. 3D) and was used for the malathion experiments. This operating point provided rapid on-demand release under the tested assay conditions while avoiding the higher temperatures required by some thermally actuated valve technologies.

#### 3.1.4. Effect of storage on retention and actuation time

Freshly prepared valves and valves stored for one week under refrigerated and ambient conditions remained closed throughout the 35 min observation period (Fig. 4). All devices used in the storage study were fabricated in the same production batch and subsequently divided into independent subsets for each storage condition and evaluation time point. Devices were sealed in zip bags and stored either under ambient conditions (20 ± 2 °C, approximately 50–60% RH) or under refrigerated conditions at 4 °C. Longer storage resulted in variable retention performance. After one month, retention decreased to approximately 14 min under refrigerated storage and 5 min under ambient storage. After two months, retention times of approximately 19 and 16 min were observed under refrigerated and ambient conditions, respectively.

**Fig. 4:**
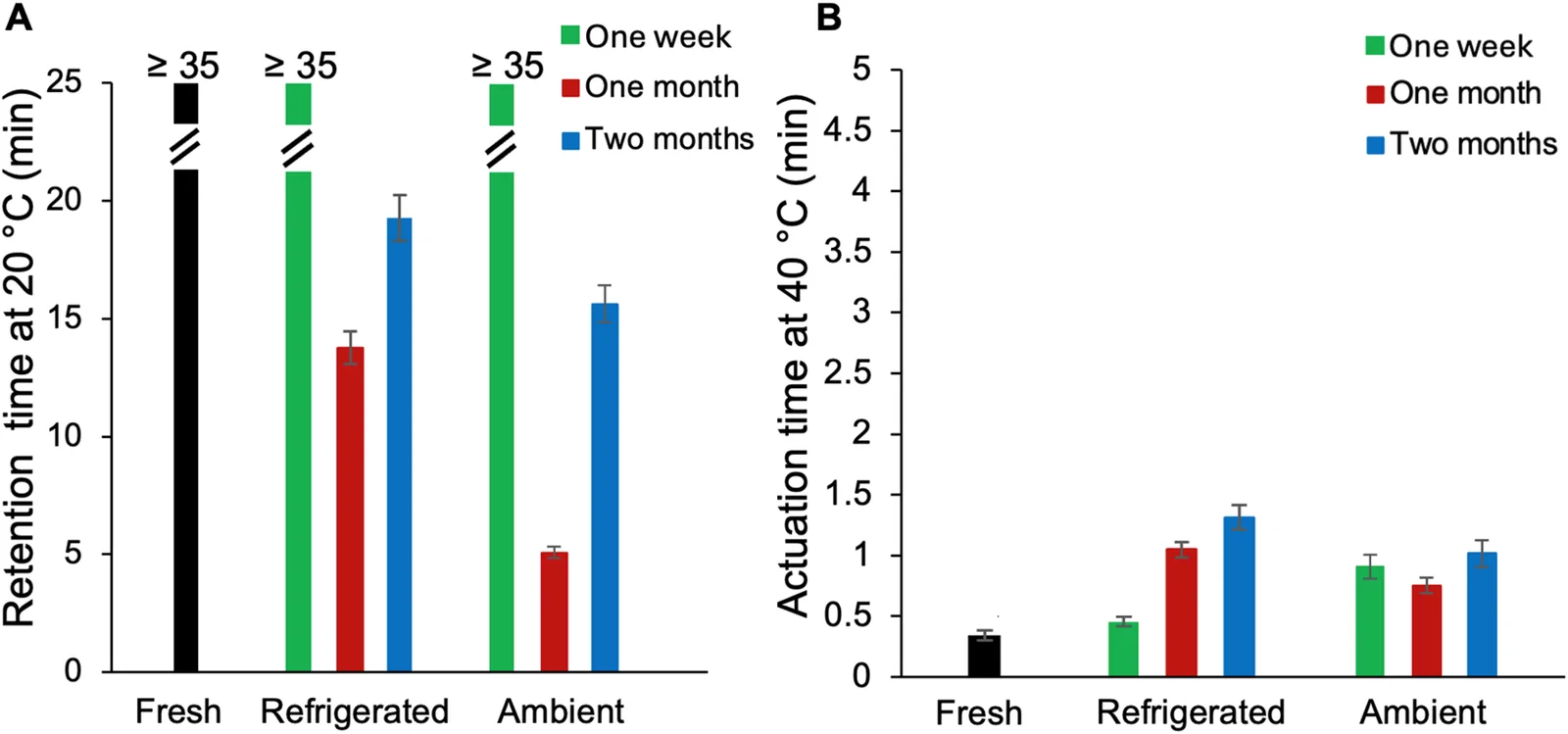
Effect of storage condition and duration on gelatin-valve performance. (A) Closed-state retention time measured at 20 °C for freshly prepared valves and valves stored at 4 °C or under ambient conditions (20 ± 2 °C) for one week, one month, and two months. Values labelled ≥35 min indicate that no passage was observed during the 35 min observation period and the sample dried out. (B) Actuation time measured at 40 °C after storage under the same conditions. Data are presented as mean ± SD (*n* = 4).

The apparent increase between one and two months should not be interpreted as progressive improvement during storage. Because all devices originated from the same production batch, the difference between these time points cannot be attributed to fabrication-lot variation. The relatively small dispersion within each storage group indicates consistent behavior among devices tested under the same condition. However, relative humidity under refrigerated storage and within the zip bags was not independently monitored or controlled. Therefore, the effects of temperature and moisture exchange cannot be separated from the present data. The non-monotonic retention behavior is consequently interpreted as evidence that long-term closed-state performance is sensitive to storage history and environmental conditions rather than as a defined aging trend.

Despite variation in closed-state retention, all stored valves remained thermally responsive and opened within approximately 1.5 min at 40 °C. These findings indicate that thermal actuation was preserved more consistently than sealing. Accordingly, the present results demonstrate reliable short-term storage and preservation of thermal responsiveness over two months, while also identifying the need for improved packaging and environmental control before establishing a definitive long-term shelf life.

### 3.2. Contact angle measurement

Contact angle measurements were used to compare untreated paper, the closed gelatin-valve region, and the valve region after thermal actuation and fluid passage (Fig. 5A–C).

**Fig. 5:**
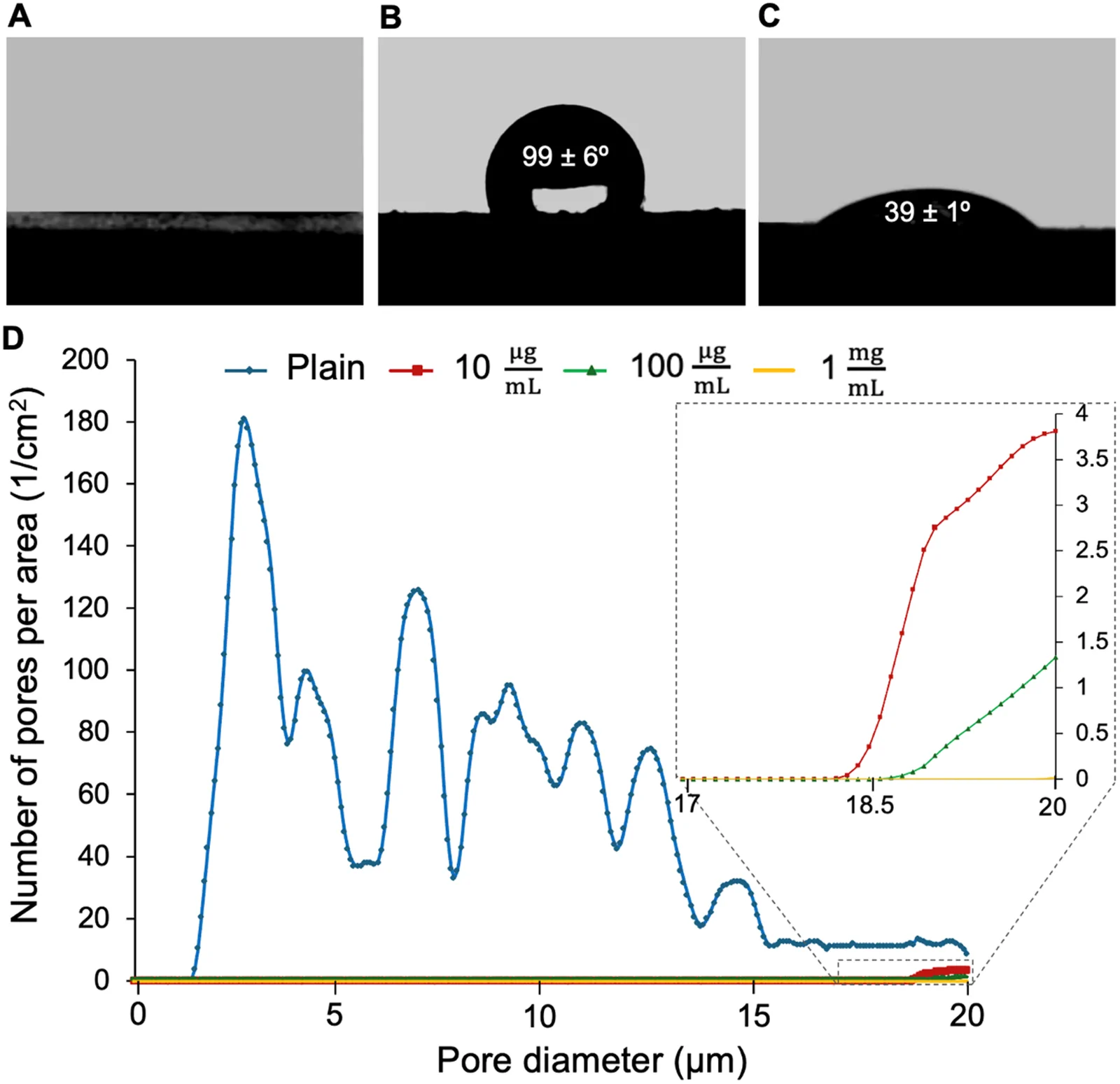
Water contact angle and porometry measurements. (A) Untreated paper, (B) closed gelatin-valve region, and (C) valve region after thermal actuation and fluid passage. (D) Accessible through-pore distribution of Whatman grade 1 paper before and after gelatin treatment. The untreated-paper curve uses a substantially larger scale (0–200 cm^−2^) than the gelatin-treated samples (0–4 cm^−2^). The inset enlarges the 17–20 µm region for the gelatin-treated samples; the untreated-paper value remains above the inset range. The data therefore show a reduction in accessible continuous pore pathways across the measured diameter range after gelatin treatment.

Untreated paper rapidly absorbed the droplet and did not sustain a measurable static contact angle. In contrast, the closed gelatin-valve region exhibited a contact angle of 99 ± 6°, indicating reduced wettability and an increased barrier to spontaneous aqueous wicking.

After thermal actuation and fluid passage, the contact angle decreased to 39 ± 1°. The treated region therefore became sufficiently wettable to support capillary transport, although it did not recover the near-instantaneous wetting behavior of untreated paper, which may reflect residual gelatin associated with the cellulose fibers. The difference between the closed and actuated states indicates that local wettability plays an important role in valve operation. The high contact angle in the closed state suppresses spontaneous capillary advance, whereas the lower contact angle after actuation is consistent with restored wetting and fluid transport.

### 3.3. Pore-structure and morphological characterization

The pore size distribution of the filter paper at varying gelatin concentrations was quantified. Fig. 5D shows that the untreated Whatman grade 1 paper exhibits a broad pore size distribution with a significant density of interfiber voids in the range of 0 to 20 µm. The peak pore density for the plain paper reached approximately 180 pores per cm^2^, confirming the highly porous nature of the cellulose network that facilitates capillary flow.

Upon the addition of gelatin, a drastic reduction in flow-permissive porosity was observed. As shown in the inset of Fig. 5D, even low concentrations (10 and 100 µg mL^−1^) significantly reduced the population of measurable pores, shifting the distribution to the upper detection limit. Most notably, the sample containing 1 mg mL^−1^ gelatin produced an essentially flat pore-distribution profile, with no resolvable flow-permissive through-pore signal over the measured range. Note that gelatin did not create additional large pores. The apparent increase in the 17–20 µm region arose from enlargement of the low-count gelatin-treated data, whereas the untreated paper retained a larger accessible-pore count in the same range. Porometry detects continuous flow-permissive pathways; therefore, the reduction in measured pore count indicates a reduction in accessible connected flow pathways rather than uniform contraction of every interfiber void.

Gelatin concentrations up to 1 mg mL^−1^ were used for quantitative porometry because preliminary measurements at higher concentrations produced essentially flat pore-distribution profiles with no resolvable through-pore signal over the measured range. The lower-concentration series therefore allowed differences in accessible connected pathways to be distinguished.

Capillary pressure can be described by the Young–Laplace relationship, 
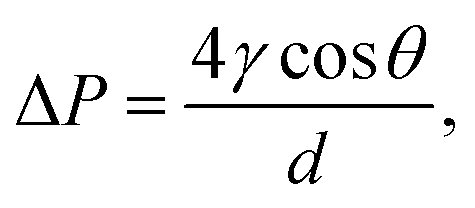
 where Δ*P* is the capillary pressure, *γ* is the liquid surface tension, *θ* is the contact angle, and *d* is the effective pore diameter. The porometry results indicate a reduction in accessible connected pore pathways, while the contact angle of approximately 99° makes cos *θ* negative. Together, these observations indicate that gelatin treatment alters both accessible capillary pathways and local wettability in a manner that opposes spontaneous wicking. However, these measurements do not establish which contribution dominates valve closure.

SEM analysis was performed to examine whether gelatin deposition produced morphological changes in the cellulose network. Representative images showed that the overall fibrous architecture and open interfiber structure were retained after gelatin treatment and thermal actuation. Although localized film-like deposits and bridging structures could be observed in some gelatin-treated regions, the differences were heterogeneous and not sufficiently pronounced for quantitative interpretation (Fig. S3). Additional SEM imaging was performed on paper treated with 45 mg mL^−1^ gelatin before and after thermal actuation (Fig. S3E and F), representing a higher concentration than the 30 mg mL^−1^ formulation used for the operational valve. Even at this higher concentration, the cellulose-fiber network remained clearly visible, and no pronounced large-scale filling of the interfiber voids was observed.

Taken together, the contact-angle measurements, porometry results, SEM observations, and fluid-flow behavior indicate that valve operation is associated with altered local wettability and modification of accessible capillary pathways following gelatin deposition. The high contact angle of the closed valve suppresses spontaneous wetting, while the porometry results demonstrate a reduction in accessible connected flow pathways. SEM observations indicate that these effects occur without complete filling of the visible interfiber structure. Accordingly, the present results do not establish localized pore-throat obstruction or complete pore filling as the sole or dominant mechanism of valve closure.

### 3.4. Application in malathion detection

The assay relies on AChE-catalyzed hydrolysis of colorless IDA, followed by formation of a blue indigo product (Fig. 2A). In the presence of organophosphates such as malathion, AChE activity is inhibited, resulting in a reduction of blue color intensity inversely proportional to the pesticide concentration.^38^ The gelatin valve separates the 15 min enzyme-inhibition step from subsequent substrate delivery, preventing IDA from reaching AChE before the incubation period is complete.

Two configurations were evaluated: a manually operated double-shot design and an operationally streamlined single-shot design (Fig. 2B and C). The devices were functionalized and operated as described in Section 2.6.

The single-shot design incorporated a bypass channel parallel to the main flow path. The main channel was 1.5 mm wide, the bypass was 1 mm wide, and the detection zone was 3 mm in diameter. This geometry allowed the sample to reach and rehydrate the AChE-containing detection zone while the gelatin valve remained closed, thereby separating sample delivery from subsequent IDA release.

The double-shot configuration produced a concentration-dependent malathion response (Fig. S4). Its four-parameter logistic fit yielded an IC_50_ of 11.3 µM and a calculated blank-based LOD of 62 nM. These results verify that the gelatin valve can support temporally separated inhibition and substrate-delivery steps.

The double-shot configuration was evaluated as an initial reference architecture for demonstrating valve-mediated temporal separation of the inhibition and substrate-delivery steps. This configuration has a relatively simple geometry and requires only 2 µL of sample; however, it also requires a second manual addition of 7 µL water after the 15 min inhibition period. Its principal role in this study is therefore as a reference configuration rather than as the preferred integrated analytical workflow. In contrast, the single-shot configuration uses a bypass channel to deliver a 15 µL sample to the AChE-containing detection zone while the gelatin valve isolates the substrate. After the defined inhibition period, thermal actuation releases the substrate without an additional liquid-addition step. The single-shot architecture therefore provides greater operational integration and was selected as the preferred configuration for subsequent analytical evaluation.

In the single-shot configuration, one 15 µL sample addition delivered the sample through the bypass channel to the AChE-containing detection zone. After the 15 min inhibition period, controlled heating at 40 °C opened the gelatin valve and released IDA. Thus, the single-shot architecture reduced the number of liquid-addition steps while retaining the same underlying inhibition chemistry. Future work may replace the benchtop hot plate with a compact controlled heater. The complete operating sequences for the double-shot and single-shot configurations, including sample addition, incubation, substrate delivery, and thermal valve actuation, are summarized schematically in Fig. 6.

**Fig. 6:**
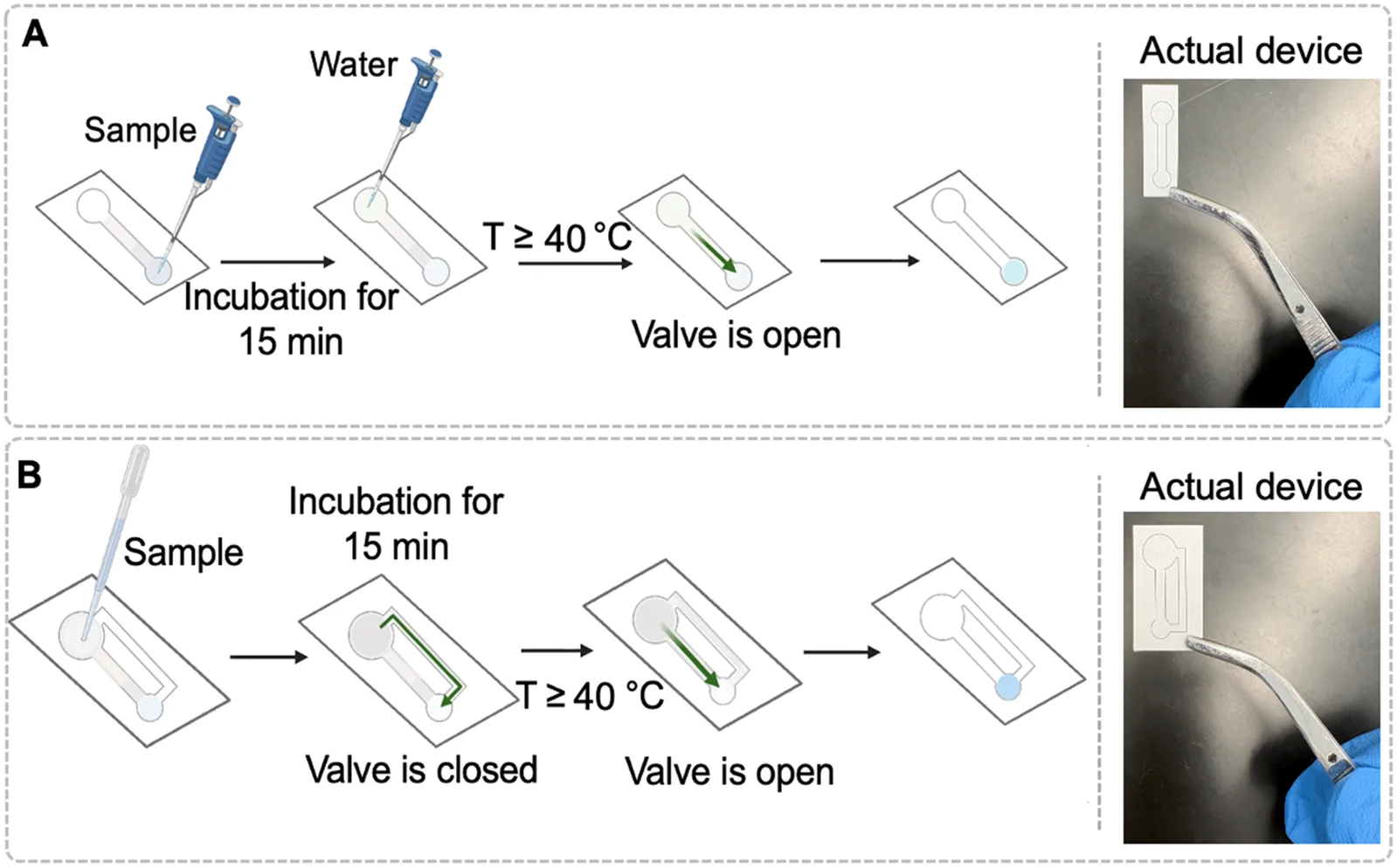
Malathion-assay workflows. (A) Double-shot: 2 µL sample, 15 min incubation, 7 µL deionized water, and controlled heating at 40 °C. (B) Single-shot: one 15 µL sample addition, 15 min incubation, and controlled heating at 40 °C. Green arrows (→) indicate fluid transport; Parafilm^®^ lamination limits leakage beneath the paper.

The single-shot device was evaluated using malathion concentrations from 10 nM to 10 mM (Fig. 7). Increasing malathion concentration progressively reduced blue color intensity. The fitted four-parameter logistic equation was 
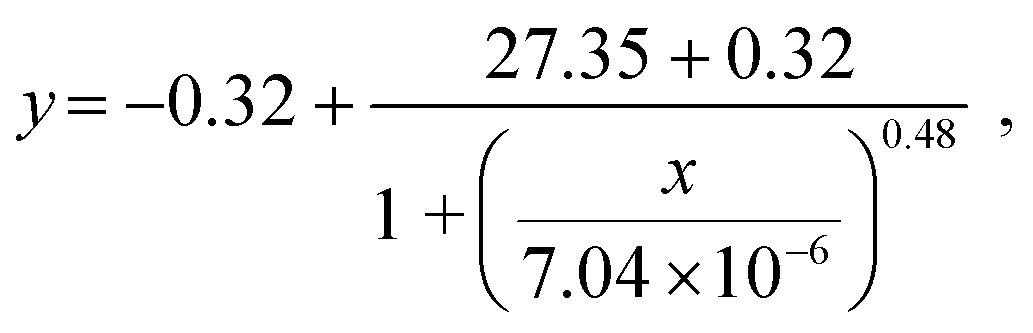
 where *x* is the malathion concentration in mol L^−1^. The fit gave *R*^2^ = 0.985 and IC_50_ = 7.041528 × 10^−6^ M, corresponding to approximately 7.04 µM.

**Fig. 7:**
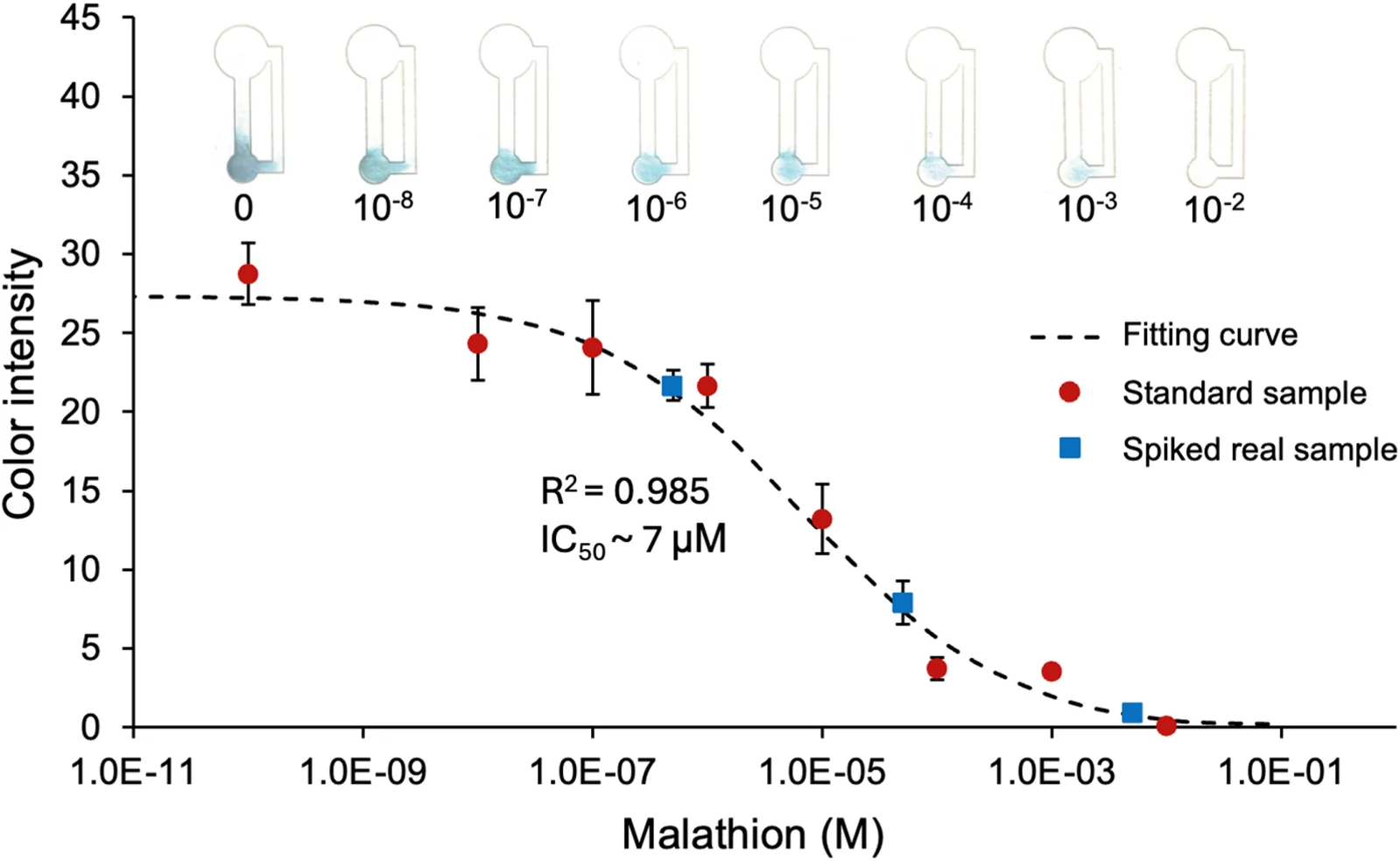
Malathion dose–response obtained using the single-shot paper-based device. Red circles (●) represent standards prepared in Tris buffer, and blue squares (■) represent malathion-spiked St. Lawrence River samples. Malathion standards from 10 nM to 10 mM were evaluated. Inset photographs show the decrease in blue color intensity with increasing malathion concentration (mean ± SD; *n* = 6).

For the single-shot device, the blank-based response threshold was 22.85. Inverse prediction from the fitted curve gave an LOD of 2.4 × 10^−7^ M, corresponding to 0.24 µM. The device was experimentally evaluated using malathion standards from 10 nM to 10 mM. Although measurable responses were obtained below 1 µM, a formal limit of quantification was not independently established. Therefore, the present study reports the calculated LOD and experimentally evaluated concentration range without assigning a formal quantitative working range. Concentrations at or above 1 mM approached the maximum-inhibition plateau and were treated as part of the saturation region.

Health Canada has established a maximum acceptable malathion concentration of 0.29 mg L^−1^ in drinking water, corresponding to approximately 0.88 µM.^42^ The calculated single-shot LOD of 0.24 µM is below this value. In addition, a 0.5 µM malathion spike in St. Lawrence River water produced a measurable response with a recovery of 86.1 ± 3.2%, demonstrating analytical response below the previously stated 1 µM boundary and within the concentration region relevant to the drinking-water guideline. However, the present study was not intended as a regulatory compliance validation. The current device is therefore positioned as a proof-of-concept screening platform, with additional matrix validation required before regulatory drinking-water analysis.

Exploratory matrix testing was performed using St. Lawrence River water spiked at 0.0005, 0.05, and 5 mM. Recoveries of 86.1 ± 3.2%, 74.9 ± 9.5%, and 81.3 ± 4.2%, respectively, showed that the device retained a concentration-dependent response in river water, but the lower-than-100% values indicate moderate matrix suppression. Notably, the 0.5 µM spike produced a measurable response below the previously stated 1 µM boundary. These measurements are therefore interpreted as exploratory evidence of matrix compatibility rather than full quantitative validation.

### 3.5. Scalability and sequential fluid control

While the single-shot malathion assay demonstrates a two-step biochemical process, the gelatin valve can also be integrated into device architectures containing multiple independently addressable fluidic pathways. To evaluate operation beyond an isolated single-valve configuration, prototype devices were fabricated for parallel and sequential fluid delivery.

Video S1 provides a qualitative demonstration of simultaneous fluid release through four parallel channels, whereas Video S2 demonstrates ordered actuation in a radial multi-valve configuration. These experiments demonstrate two fluid-control functions relevant to multistep paper-based assays: simultaneous operation of multiple channels and temporally ordered release from individual channels.

Two distinct spacing constraints should be considered. During fabrication, 0.5 µL of gelatin produced a treated region approximately 5 mm long in the direction of flow; therefore, the valve footprints and sample-loading zones must remain physically separated. In the parallel device, the transverse edge-to-edge separation between adjacent valve regions was 5 mm, and no visually apparent overlap was observed under the tested conditions.

During sequential operation, heat was applied manually and locally to the selected valve region. Only the targeted valve visibly opened, while the remaining valves remained closed until they were heated individually. This observation supports independent localized actuation for the tested geometry. However, smaller transverse separations and the temperatures at neighboring valves were not systematically evaluated.

The parallel and sequential devices should be regarded as fluidic proof-of-concept demonstrations rather than complete analytical assays. Their purpose is to demonstrate fluid-control operations that may be useful in paper-based multistep assays requiring controlled reagent delivery, incubation, or washing. The malathion sensor provides a separate biochemical proof-of-concept in which the gelatin valve temporally separates the AChE-inhibition step from subsequent substrate delivery. A complete immunoassay, such as an ELISA, was not implemented in the present study and would require assay-specific integration and validation.

### 3.6. Comparison with existing valves

Table 1 compares the proposed gelatin valve with representative active and passive flow-control strategies used in paper microfluidics. The comparison focuses on active or passive operation, triggering conditions, reported response or delay time, and major practical considerations. A PNIPAM-based thermoresponsive valve is included because its low-temperature thermal response provides a relevant comparison with the gelatin valve.

**Table 1:** Comparison of flow control strategies in µPADs

Valve material	Active/passive	Trigger and operating condition	Actuation time	Main practical considerations	Reference
Gelatin valve	Active	Heating at 40 °C; 30 mg mL^−1^ gelatin	∼20 s	Simple deposition without polymer synthesis; refrigerated performance demonstrated for two months, while ambient-storage retention was variable	This work
Wax valve	Active	Local heating; 41 °C on cellulose	∼15 s	Thermally reversible and capable of repeated actuation; requires wax printing and a localized thin-film heater	11
Shape-memory polymer valve	Active	Joule heating above the polymer transition temperature; approximately 95 °C	∼22.7 s to open; ∼24.4 s to close	Reversible two-way switching and automated sequential delivery; requires an SMP actuator, paper cantilever, resistive heater, PCB, and control electronics	12
PNIPAM-coated membrane	Active	Temperature change across approximately 32 °C	NR	Reversible low-temperature switching; requires plasma-induced graft polymerization onto a porous membrane	21
Dissolvable polymer barrier	Passive	Contact with aqueous fluid	Minutes; material- and geometry-dependent	Simple operation without external actuation; humidity sensitivity depends on the material and formulation	18 and 19

The gelatin valve provides active fluid control at 40 °C without requiring polymer synthesis, surface grafting, or integrated mechanical components. Under the optimized condition, fluid traversed the valve in approximately 20 s. Functional re-closing after fluid passage was not demonstrated, and longer-term closed-state retention varied with storage condition. PNIPAM valves offer more clearly demonstrated reversible switching but require graft polymerization. Reported response times should be interpreted cautiously because the cited studies used different geometries and definitions of valve opening; NR denotes that no directly comparable trigger-to-opening time was reported.

## 4 Conclusion

This study demonstrates thermo-responsive gelatin valves for timed fluid control in µPADs. A 30 mg mL^−1^ gelatin formulation cooled at 4 °C for 15 min provided closed-state retention compatible with the 15 min malathion assay and opened in approximately 20 s under controlled heating at 40 °C. Integration into a single-shot AChE inhibition assay produced a calculated LOD of 0.24 µM. A measurable response was also obtained for a 0.5 µM malathion spike in St. Lawrence River water. Although the calculated LOD is below the guideline value of approximately 0.88 µM and the device retained a concentration-dependent response in spiked river water, additional matrix validation and improved recovery are required before compliance-level drinking-water analysis.

The gelatin valve provides active, user-defined flow control, whereas dissolvable barriers provide passive timing without an external trigger. Environmental sensitivity depends on material formulation and storage conditions for both approaches. The gelatin valves retained thermal responsiveness for two months, although closed-state retention varied during longer storage and humidity was not independently controlled. Functional re-closing after fluid passage was not demonstrated, so the present device is considered single-use. The maximum ambient operating temperature and performance after thermal cycling also remain to be established. Future work will focus on controlled-humidity testing, moisture-resistant packaging, compact localized heaters, thermal-cycling studies, and assay-specific integration into multiplexed workflows.

## Conflicts of interest

The authors declare no competing financial interest.

## Supplementary Material

RA-016-D6RA07964D-s001

RA-016-D6RA07964D-s002

RA-016-D6RA07964D-s003

## Data Availability

All data generated or analyzed during this study are included in this published article and its supplementary information (SI). Supplementary information: laser-cutting parameters, additional data on the effects of cooling conditions and gelatin concentration on valve performance, SEM micrographs, double-shot malathion assay data, and descriptions of supplementary videos demonstrating parallel and sequential valve operation. See DOI: https://doi.org/10.1039/d6ra07964d.
