# Supplementary material for: Stopping, opening, and redirecting fluid flow in paper-based microfluidic devices using thermo-responsive gelatin valves
Source: RSC Adv. 2026 Oct 5;16(46):60239–49. doi: 10.1039/d6ra07964d (PMC13637522; doi:10.1039/d6ra07964d)
Supplement: Supplementary file 1 [file RA-016-D6RA07964D-s001.pdf]

Supplementary Information (SI) for RSC Advances.
This journal is © The Royal Society of Chemistry

Supporting Information

Stopping, Opening, and Redirecting Fluid Flow in Paper-Based Microfluidic Devices Using Thermo-Responsive Gelatin Valves

Abdolali Mehrjou, Seyed Hamid Safiabadi Tali, Mohsen Ghiasi Tarzi, Zubi Sadiq, Sana Jahanshahi-Anbuhi*

Department of Chemical and Materials Engineering, Gina Cody School of Engineering,

Concordia University, Montréal, Québec H4B 1R6, Canada

* Address correspondence to Sana Jahanshahi-Anbuhi, sana.anbuhi@concordia.ca

Table of Contents

SI 1. Laser settings 2

SI 2. Effect of cooling time and temperature 2

SI 3. Effect of gelatin concentration 5

SI 4. SEM micrographs 7

SI 5. Double-shot configuration 9

SI 6. Supplementary videos 10

SI 1. Laser settings

A CO_2 laser cutter (VLS 2.30DT, Universal Laser Systems; 30 W, 2.0 lens) was utilized to pattern the microfluidic channels onto Whatman grade 1 filter paper. The device designs were created using Inkscape software (version 1.1.1). Table S1 details the specific laser parameters used for cutting the device components.

Table S1. Laser settings used for device fabrication.

Laser power (%)	Laser speed (%)	Z-axis distance (inches)	Pulses per inch (PPI)	Mode
85	100	0.014	500	Vector

SI 2. Effect of cooling time and temperature

To optimize the valve fabrication protocol, we evaluated the impact of cooling conditions on the structural integrity of the gelatin network. Devices containing 30 mg/mL gelatin valves were subjected to gelation at either ambient temperature (20 ± 2 °C) or refrigerated conditions (4 °C) for varying durations (5, 10, and 15 minutes). Following gelation, 7 μL of blue-dyed water was introduced to the inlet, and fluid permeation was monitored over time.

As shown in Figure S1, the cooling temperature was the dominant factor governing valve performance. Valves gelled at room temperature (Panels A-C) failed to form a sufficiently dense network, resulting in fluid leakage within 3-4 minutes regardless of the cooling duration (5-15 min). This rapid failure renders ambient cooling unsuitable for the 15-minute incubation required by the assay.

Refrigerated cooling improved retention time; however, only the 15 min condition met the predefined criterion that all four independently prepared valves remain closed throughout the 35 min observation

period (Panels D-F). Therefore, cooling at 4 °C for 15 min was selected as the standard fabrication protocol.

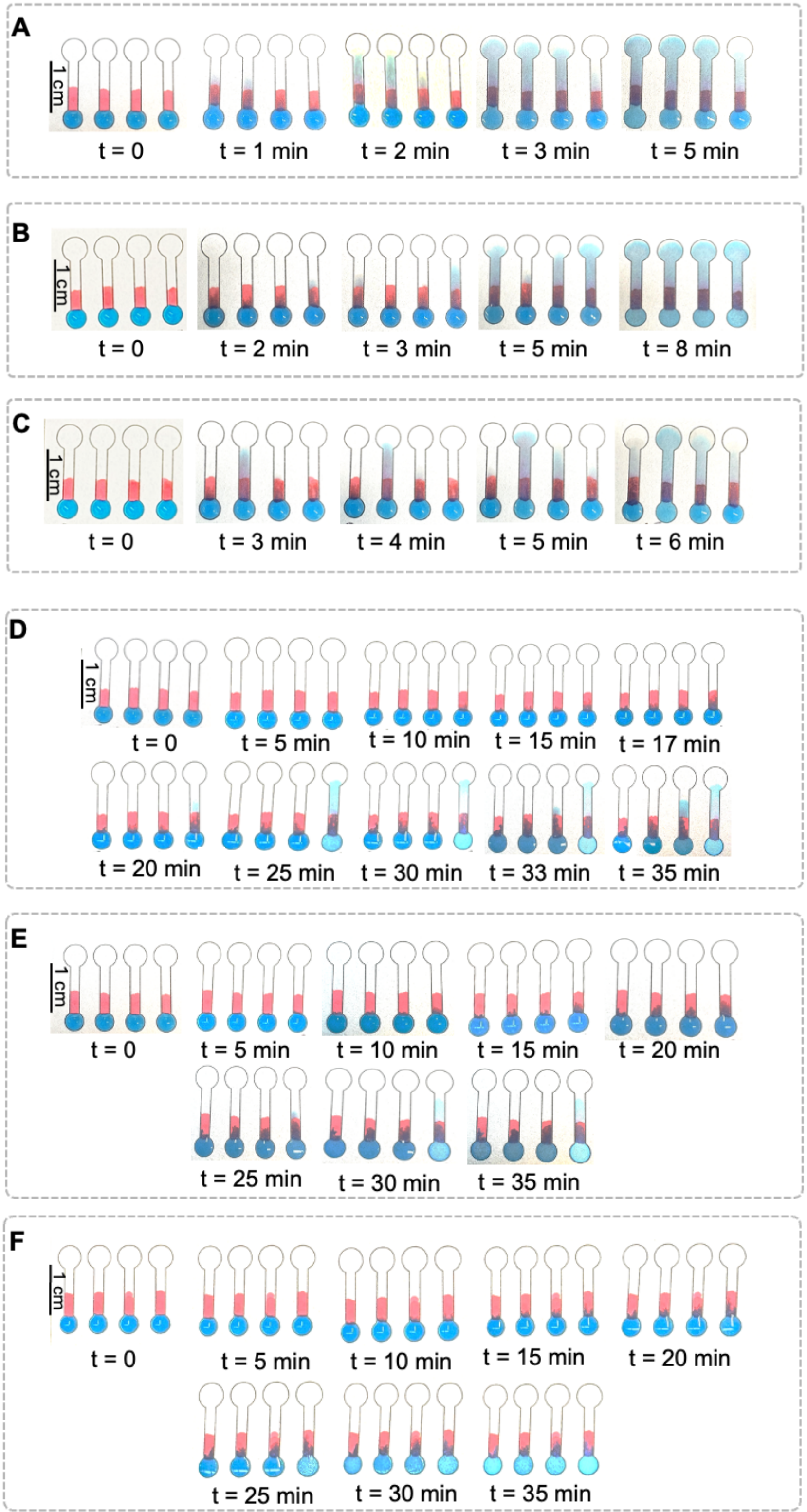

Figure S1. Time-lapse comparison of cooling conditions on gelatin valve sealing. Valves (30 mg/mL) were cooled down under the following conditions: **(A)** Ambient air (20 °C) for 5 min. **(B)** Ambient air

(20 °C) for 10 min. **(C)** Ambient air (20 °C) for 15 min. **(D)** Refrigerator (4 °C) for 5 min. **(E)** Refrigerator (4 °C) for 10 min. **(F)** Refrigerator (4 °C) for 15 min.

SI 3. Effect of gelatin concentration

To determine the critical polymer density required for effective fluid gating, valves were fabricated using a range of gelatin concentrations (10-45 mg/mL) and subjected to a water retention test. The sealing capability was assessed by adding 7 μL of blue-dyed water to the inlet and recording the fluid permeation over a 35-minute observation window.

To form the initial thermal barrier, the deposited gelatin solution was gelled at 4 °C for 15 minutes. As illustrated in Figure S2, a distinct transition in sealing performance was observed between 20 and 30 mg/mL. Valves with low gelatin concentrations (Panels A and B) exhibited insufficient network density to resist capillary pressure. Specifically, the 10 mg/mL valves failed almost immediately, with fluid breaching the barrier within 5-8 minutes. Similarly, the 20 mg/mL formulation slowed the flow but eventually allowed fluid passage, rendering it unsuitable for the 15-minute incubation step required by the assay.

In contrast, increasing the concentration to 30 mg/mL (Panel C) resulted in a mechanically robust seal capable of containing the fluid for the full 35-minute duration without leakage. Higher concentrations (35-45 mg/mL, Panels D-F) demonstrated comparable sealing efficiency; however, as noted in the main text, these denser networks required longer heating times for activation. Therefore, the 30 mg/mL formulation was identified as the optimal concentration, providing the necessary sealing integrity while minimizing the thermal energy required for actuation.

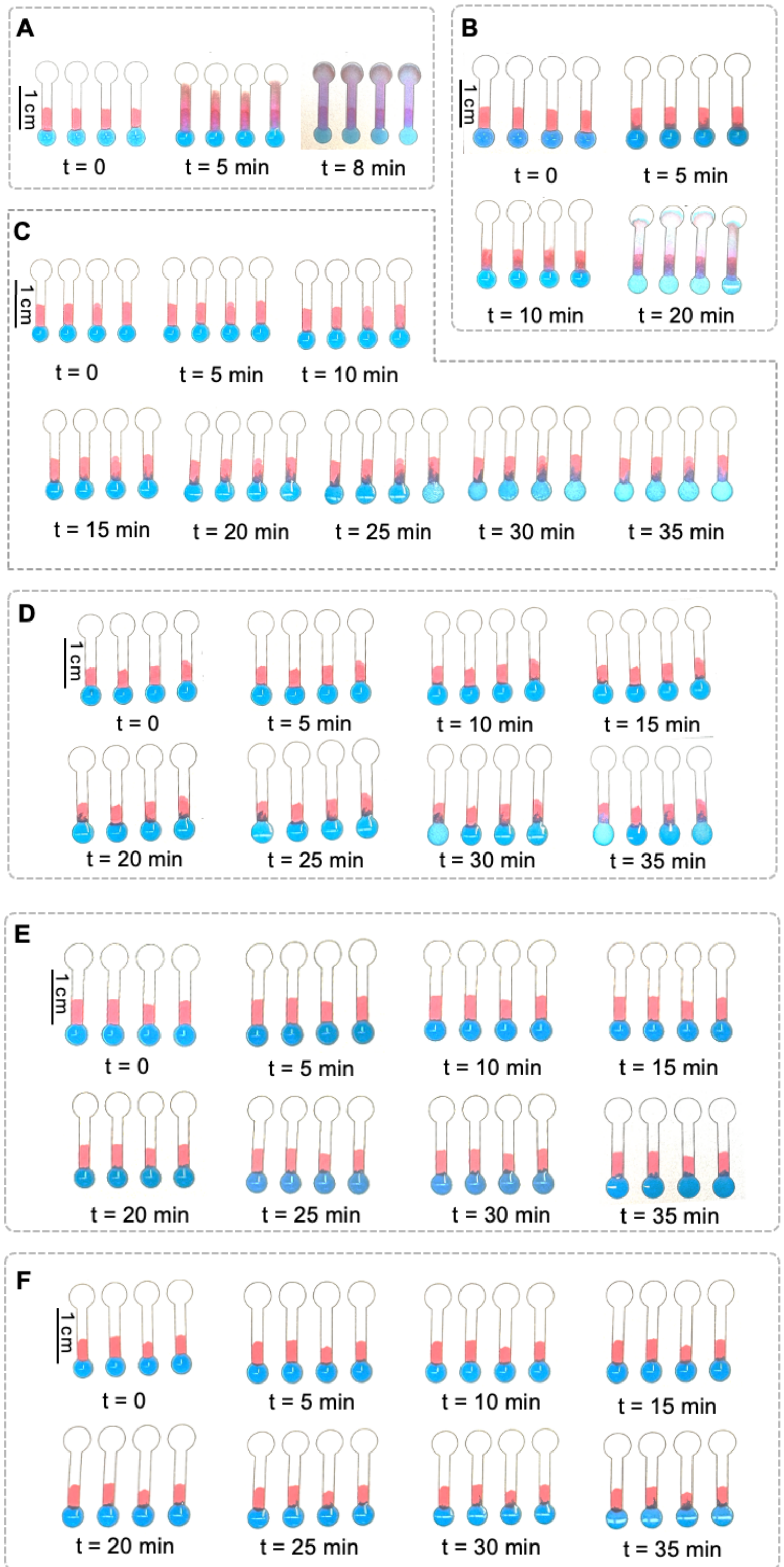

Figure S2. Photographic evaluation of gelatin concentration. Fluid penetration was monitored for valves prepared with **(A)** 10, **(B)** 20, **(C)** 30, **(D)** 35, **(E)** 40, and **(F)** 45 mg/mL gelatin. The deposited gelatin was gelled at 4 °C for 15 min before testing. Under the tested conditions, concentrations of 30 mg/mL

or greater prevented visible fluid passage throughout the 35 min observation period required to cover the malathion incubation step.

SI 4. SEM micrographs

To further examine the interaction between gelatin and the cellulose fiber network, SEM imaging was performed on untreated paper, the gelatin-valve region before and after thermal actuation, and the downstream detection zone (Figure S3). The overall fibrous structure of the paper remained visible under all conditions, indicating that gelatin deposition and thermal actuation did not cause major structural deformation of the cellulose matrix. Subtle localized deposits and fiber-bridging features were observed in the gelatin-treated regions; however, the morphological differences were heterogeneous and not pronounced at this magnification. These images therefore provide qualitative support for localized gelatin integration within the paper network rather than evidence of complete pore filling.

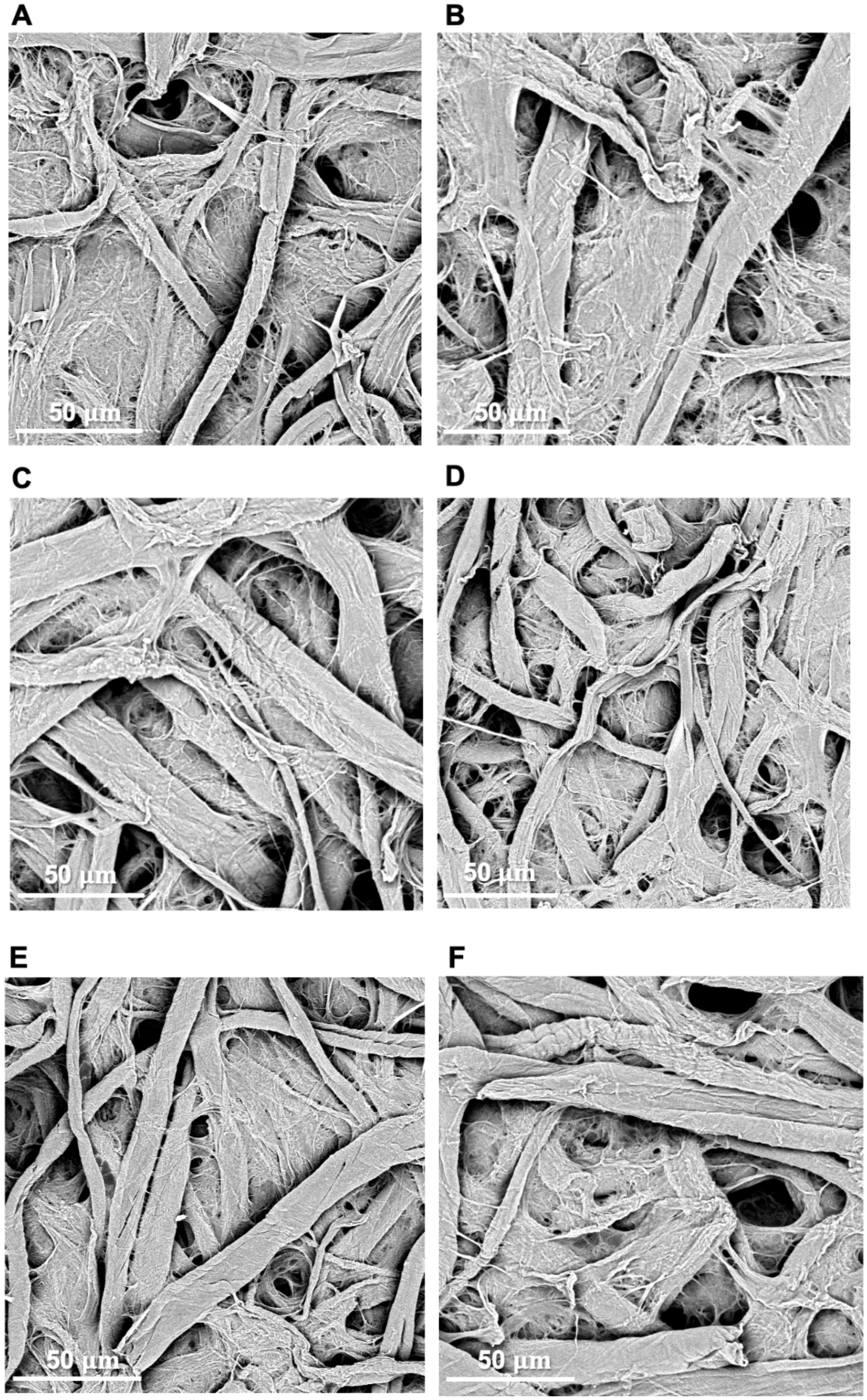

Figure S3. Representative SEM images of the paper and gelatin-valve regions. **(A)** Untreated Whatman grade 1 filter paper, **(B)** gelatin-treated valve region before thermal actuation, **(C)** gelatin-treated region after thermal actuation and fluid passage, and **(D)** downstream detection zone after valve actuation and arrival of the fluid. Additional images of paper treated with 45 mg/mL gelatin before and after thermal

actuation are shown in panels **(E)** and **(F)**, respectively. Even at the higher gelatin concentration, the cellulose-fiber network remained visible and no pronounced large-scale filling of the interfiber voids was observed. Localized coating and bridging features were present in some regions. Scale bars are equal to 50 μm.

SI 5. Double-shot configuration

The double-shot configuration was evaluated using malathion standards and spiked St. Lawrence River samples (Figure S4). Increasing malathion concentration decreased the blue color intensity, consistent with concentration-dependent AChE inhibition. The four-parameter logistic fit was $y = -1.61 + \frac{31.28 + 1.61}{1 + \left(\frac{x}{1.13 \times 10^{-5}}\right)^{0.42}}$, where x is the malathion concentration in mol/L. The fit yielded $R^2 = 0.982$ and IC_{50} = 1.12939 × 10⁻⁵ M, corresponding to approximately 11.3 μM. Using a blank-based response threshold of 27.97 and inverse prediction from the fitted curve, the calculated LOD was 6.2 × 10⁻⁸ M, corresponding to 62 nM.

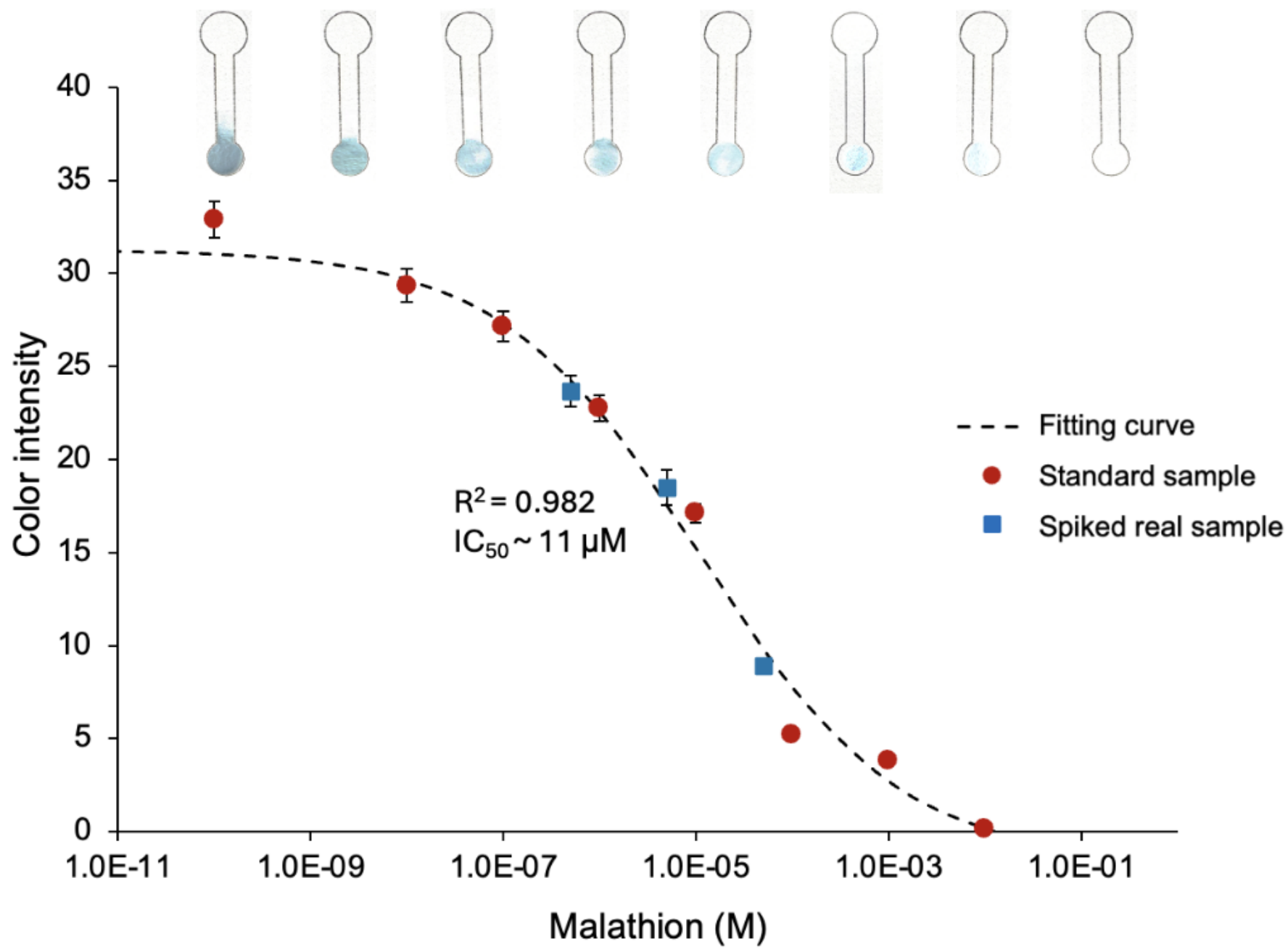

Figure S4. Malathion dose-response obtained using the double-shot paper-based device. Red circles represent standards prepared in Tris buffer, blue squares represent malathion-spiked St. Lawrence River samples, and the dashed line represents the four-parameter logistic fit to the standard calibration data (R^2 = 0.982; IC_{50} = 11.3 μM). The blank-based calculated LOD was 62 nM. Inset photographs show the decrease in blue color intensity with increasing malathion concentration (mean ± SD; n = 6).

SI 6. Supplementary videos

Video S1. Parallel operation of gelatin valves. Simultaneous actuation of gelatin valves integrated into four parallel paper-fluidic channels. The video is a qualitative demonstration of synchronized multi-channel fluid delivery.

Video S2. Sequential operation of gelatin valves. Localized manual heating was applied to individual valves in a radial multi-valve device. Only the targeted valve visibly opened, while the remaining valves stayed closed until separately heated. The video is a qualitative demonstration of ordered fluid delivery rather than a quantitative thermal-crosstalk study.
